# Cyanobacteria in Sulfidic Spring Microbial Mats Can Perform Oxygenic and Anoxygenic Photosynthesis Simultaneously during an Entire Diurnal Period

**DOI:** 10.3389/fmicb.2016.01973

**Published:** 2016-12-15

**Authors:** Judith M. Klatt, Dirk de Beer, Stefan Häusler, Lubos Polerecky

**Affiliations:** ^1^Microsensor Group, Max-Planck-Institute for Marine MicrobiologyBremen, Germany; ^2^Geomicrobiology Lab, Department of Earth and Environmental Sciences, University of Michigan, Ann ArborMI, USA; ^3^Department of Earth Sciences – Geochemistry, Faculty of Geosciences, Utrecht UniversityUtrecht, Netherlands

**Keywords:** microbial mat, cyanobacteria, anoxygenic photosynthesis, sulfide:quinone:reductase, hydrogen sulfide, microsensors

## Abstract

We used microsensors to study the regulation of anoxygenic and oxygenic photosynthesis (AP and OP, respectively) by light and sulfide in a cyanobacterium dominating microbial mats from cold sulfidic springs. Both photosynthetic modes were performed simultaneously over all H_2_S concentrations (1–2200 μM) and irradiances (4–52 μmol photons m^-2^ s^-1^) tested. AP increased with H_2_S concentration while the sum of oxygenic and anoxygenic photosynthetic rates was constant at each light intensity. Thus, the total photosynthetically driven electron transport rate was solely controlled by the irradiance level. The partitioning between the rates of these two photosynthetic modes was regulated by both light and H_2_S concentration. The plastoquinone pool (PQ) receives electrons from sulfide:quinone:reductase (SQR) in AP and from photosystem II (PSII) in OP. It is thus the link in the electron transport chain where both pathways intersect, and the compound that controls their partitioning. We fitted our data with a model of the photosynthetic electron transport that includes the kinetics of plastoquinone reduction and oxidation. The model results confirmed that the observed partitioning between photosynthetic modes can be explained by a simple kinetic control based on the affinity of SQR and PSII toward PQ. The SQR enzyme and PSII have similar affinities toward PQ, which explains the concurrent OP and AP over an astonishingly wide range of H_2_S concentrations and irradiances. The elegant kinetic control of activity makes the cyanobacterium successful in the fluctuating spring environment. We discuss how these specific regulation mechanisms may have played a role in ancient H_2_S-rich oceans.

## Introduction

Oxygenic photosynthesis (OP) is a process where light energy is used to extract electrons from water to reduce CO_2_. The evolution of this type of photosynthesis was predated by anoxygenic photosynthesis (AP), a process that uses another compound (e.g., H_2_S) as electron donor for the reduction of CO_2_ (Blankenship, 2010). While the more ancient AP requires only one photosystem (PSI) to drive the electron flow, OP requires the combined power of two photosystems (PSI+PSII), primarily because of the high energy demand in the water splitting reaction.

OP probably evolved in cyanobacteria (Mulkidjanian et al., 2006) inhabiting microbial mat-like structures. In these systems alternative electron donors for photosynthesis such as H_2_S were likely abundant (Nisbet and Fowler, 1999; Buick, 2008) and remained available until the end of the Proterozoic (Canfield, 1998). Therefore, it has been hypothesized that AP performed by obligate anoxygenic phototrophs and cyanobacteria capable of both AP and OP (referred to as versatile cyanobacteria) was an important photosynthetic mode on a global scale before the complete oxygenation of the Earth’s atmosphere and oceans during the Neoproterozoic oxidation event (Johnston et al., 2009). It is therefore intriguing how oxygenic phototrophs were finally so successful in oxygenating Earth despite the widespread availability of electron donors for anoxygenic phototrophs and the toxic effects of H_2_S on the components of OP, especially considering that AP is a biochemically less complicated and energetically less demanding process than OP (Miller and Bebout, 2004; Klatt et al., 2015a).

**FIGURE 1:**
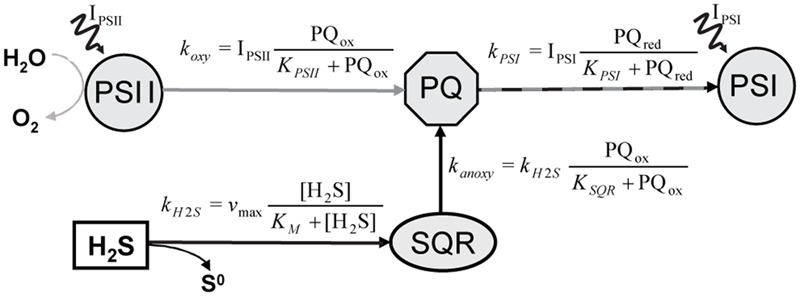
**Simplified diagram of the model describing partitioning between AP and OP in versatile cyanobacteria, as proposed by Klatt et al. (2015a).** The AP and OP pathways intersect at plastoquinone (PQ) and their partitioning is regulated by the PQ redox state. In the AP pathway, PQ is reduced in a light-independent reaction by the enzyme sulfide:quinone:reductase (SQR), which obtains the electrons from the oxidation of H_2_S to zero-valent sulfur. In contrast, PQ reduction in the OP pathway is driven by the light-dependent activity of photosystem II (PSII), with electrons originating from the oxidation of H_2_O to O_2_. In both pathways PQ oxidation is driven by the light-dependent activity of photosystem I (PSI). I_PSI_ and I_PSII_ denote light energy harvested in PSI and PSII, respectively. The rate-laws describing the kinetic regulation of the rates of OP (k_oxy_), AP (k_anoxy_), H_2_S oxidation by SQR (k_H2S_) and of the total photosynthetic electron transport (k_PSI_) are shown by the formulae.

New insights into possible mechanisms that allowed outcompetition of anoxygenic phototrophs by oxygenic phototrophs in the presence of H_2_S can be gained by studying adaptations of extant cyanobacteria living in sulfidic environments. The cold, light-exposed sulfidic springs at Frasassi, Italy (Klatt et al., 2016), which harbor thin microbial mats inhabited by diverse anoxygenic, oxygenic and versatile phototrophs, are one example of such an environment. Our recent studies of cyanobacteria isolated from this system revealed two novel cyanobacterial adaptations to H_2_S. The first adaptation mechanism, observed for a versatile cyanobacterium *Pseudoanabaena* str. FS39, included partitioning between AP and OP that is regulated kinetically by H_2_S through the apparent affinity of the electron transport components involved in AP [sulfide:quinone:reductase (SQR)] and OP [photosystem II (PSII)] toward plastoquinone, which is where both electron transport pathways intersect (**Figure 1**; Klatt et al., 2016). OP in this cyanobacterial strain is active only when the electron transport chain is not fully used by AP, which occurs when H_2_S is limiting. The second type of adaptation, observed for the obligatory oxygenic cyanobacterium *Planktothrix* str. FS34, included acceleration of the recovery of OP by H_2_S after prolonged exposure to darkness and anoxia combined with an enhancement of OP rates by H_2_S at low irradiance and a temporary resistance to H_2_S toxicity (Klatt et al., 2015b).

The microbial mats in the Frasassi sulfidic springs are characterized by a microenvironment with rapidly fluctuating availability of light and H_2_S (Klatt et al., 2016). Although both *Pseudanabaena* str. FS39 and *Planktothrix* str. FS34 have adaptations that could make them successful in such environment, microscopic observations revealed that neither of them appeared to be abundant in the system. Instead, the mats where dominated by yet another, morphologically very distinct, cyanobacterium. The aim of this study was to understand the adaptations responsible for the success of this dominant cyanobacterium in the system. We hypothesized that the dominant cyanobacterium possesses a mechanism that allows it to rapidly switch between AP and OP based on the instantaneous availability of light and H_2_S and thus be adapted to the rapidly fluctuating microenvironmental conditions in the mats. However, we expected that the specific regulation by these parameters differs from that found in *Pseudanabaena* str. FS39, given that the abundance of this strain in the mats is very low. To test this hypothesis, we quantified the combinatory effects of light and H_2_S on the partitioning between AP and OP performed by the dominant cyanobacterium. Because our cultivation attempts were not successful, we performed our measurements in a natural mat sample dominated by the cyanobacterium.

## Materials and Methods

### Microsensors

O_2_, H_2_S, and pH microsensors with a tip diameter of 10–50 μm and response time of <2 s were built, calibrated and used for profiling and monitoring of local concentration dynamics as previously described (Revsbech, 1989; Jeroschewski et al., 1996; de Beer et al., 1997; Klatt et al., 2016). A fiber-optic microprobe for scalar irradiance (Lassen et al., 1992) was used to measure the locally available light in the mat. The microprobe was connected to a spectrometer (USB4000, Ocean Optics, USA) and calibrated against a PAR scalar irradiance sensor connected to a light meter (LI-250, both from LI-COR Biosciences, Lincoln, NE, USA) as previously described (Al-Najjar et al., 2010).

### Mat Sampling and Measurement Protocol

Thin cyanobacterial mat together with the underlying sediment were sampled from the Frasassi sulfidic springs (Klatt et al., 2016) in September 2009. Immediately after sampling it was placed in an aquarium and submersed in spring water. The mat was left overnight in the dark to allow for diffusion of sulfide from the sediment into the water column and re-establishment of an anoxic sulfidic water column resembling *in situ* conditions. The conditions in the mat and water column were monitored by occasional microsensor depth profiling of O_2_, H_2_S, and pH. During measurements, the water column above the mat was covered with paraffin oil. A gentle stream of air onto the paraffin oil surface allowed for a slow, circular flow of the water column in the aquarium.

Net volumetric conversion rates of sulfide and O_2_ were calculated from the measured steady-state depth profiles of concentration, c(z), using Fick’s law of diffusion, NP = – D ∂^2^c(z)/∂z^2^, where D is the corresponding molecular diffusion coefficient corrected for temperature and salinity (Gieseke and de Beer, 2004). Specifically, we used D = 2.10 10^-9^ m^2^ s^-1^ and D = 1.59 10^-9^ m^2^ s^-1^ as the diffusion coefficients for O_2_ and sulfide, respectively. In our calculations we assumed porosity of 1, which is reasonable for a biofilm comprised primarily of microbial cells. Volumetric rates of *gross* photosynthesis in the mats were measured using the light–dark shift approach. This approach allows to differentiate between gross and net, and thus between photosynthetic and chemotrophic, sulfide and oxygen conversion rates, based on the dynamics of O_2_ and S_tot_ in the first few seconds after darkening the sample. The O_2_-based light–dark shift method is well-established and based on the dynamics of O_2_ concentration after darkening (Revsbech and Jørgensen, 1983). Analogously, gross AP rates were determined as the rate of sulfide concentration change (-dS_tot_/dt) upon darkening where the total sulfide concentration (S_tot_ = H_2_S+HS^-^+S^2-^) was calculated from the measured H_2_S concentration and pH. As described previously (Klatt et al., 2016), the variation in pH with time during a light–dark shift measurement was ignored as it was substantially slower and insignificant compared with the variation in H_2_S. During the measurements the tips of the microsensors were separated by <50 μm.

**FIGURE 2:**
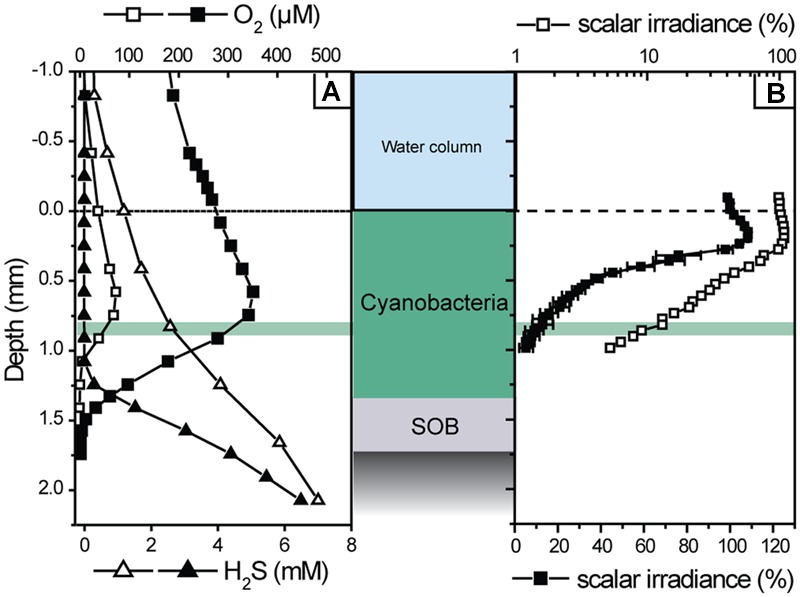
**Examples of transient depth profiles of O_2_ and H_2_S concentration (A)**, and of a depth profile of scalar irradiance **(B)**, measured in the cyanobacterial mat during illumination at incident irradiance of 169 μmol photons m^-2^ s^-1^. The profiles shown with filled symbols were measured about 2 h after those shown by open symbols. The scalar irradiance values in **(B)** were integrated over the wavelengths of photosynthetically active radiation (400–700 nm) and normalized to the value at the mat surface. Error bars represent the standard deviation of measurements in three replicate spots of the mat. Note that the filled and open symbols show the same profile in linear and logarithmic scale, respectively. The depth of highest photosynthetic activity (0.83 mm) is indicated by the green area.

First, the light–dark shift measurements were done at a constant incident irradiance (334 μmol photons m^-2^ s^-1^) at various depths of the mat to identify the depth with the highest photosynthetic activity. At this depth gross OP and AP were subsequently determined at incident irradiance levels varying between 37 and 490 μmol photons m^-2^ s^-1^, adjusted in a random order using a cold halogen light source (KL2500, Schott, Germany). During these measurements H_2_S was gradually depleted in the mat (**Figure 2A**). Therefore, the mat was occasionally incubated in the dark for up to 1 h to allow sulfide diffuse from deeper layers into the photosynthetically active zone and for the re-establishment of the initial sulfidic conditions at the depth of measurements. Occasionally, complete concentration depth profiles were measured. After the light–dark shift measurements, a depth profile of scalar irradiance was measured in the same spot to determine the locally available light. To exclude a significant contribution of potentially abundant obligate anoxygenic phototrophs to the measured rates of AP, additional measurements were performed under illumination with light emitting diodes (emission maxima at 740 and 810 nm; H2A2 series, Roithner Lasertechnik, Austria) that specifically targeted bacteriochlorophyll in the mat. Finally, a sub-sample of the measured mat was examined by microscopy using an epifluorescence microscope (Axiophot, Zeiss, Germany).

### Calculations

Total volumetric photosynthetic electron transport rates were calculated as a sum of the estimated electron transport rates due to OP and AP (Klatt et al., 2015a). The former were calculated by multiplying the gross rates of O_2_ production measured by the light–dark shift method with a factor of 4, as implied by the reaction 2H_2_O → O_2_ + 4H^+^ + 4e^-^. Analogously, the latter were calculated by multiplying the gross rates of S_tot_ depletion measured by the light–dark shift method with a factor of 2, as implied by the reaction H_2_S → S^0^ + 2H^+^ + 2e^-^.

### Model of Photosynthetic Electron Transport Chain Reactions

We used a model of the intersecting anoxygenic and oxygenic electron transport chain (**Figure 1**) to fit our data. The model is described in detail in Klatt et al. (2015a). Briefly, the model focuses on the plastoquinone pool (PQ), the central component of both electron transport pathways. In the PQ pool both pathways intersect (**Figure 1**). The ratio of the reduction rate of PQ by SQR (k_anoxy_ in **Figure 1**) and the reduction rate of PQ by PSII (k_oxy_) directly translates into the partitioning between anoxygenic and oxygenic electron transport. The parameters used for fitting the observed partitioning were thus the apparent affinities of SQR and PSII toward PQ (K_SQR_ and K_PSII_, respectively), and the affinity of SQR toward H_2_S (K_M_) and v_max_. Other parameters are not effective for the partitioning but determine the light dependency of the sum of oxygenic and anoxygenic electron transport rates.

## Results

### Mat Structure and Microscopy

Along the flow path of the sulfidic water emerging from the Frasassi Cave system structurally and functionally diverse microbial mats form (Klatt et al., 2016). In this study, we sampled a mat that was characterized by a green cyanobacterial layer on top of a distinct, very thin layer of large sulfur oxidizing bacteria (SOB). After transfer to the aquarium the water column above the mat was temporary oxic and, when the mat was left in the dark, we observed migration of SOB filaments to the surface of the mat. Exposure to an anoxic and sulfidic water column overnight led to re-establishment of the initial mat structure.

**FIGURE 3:**
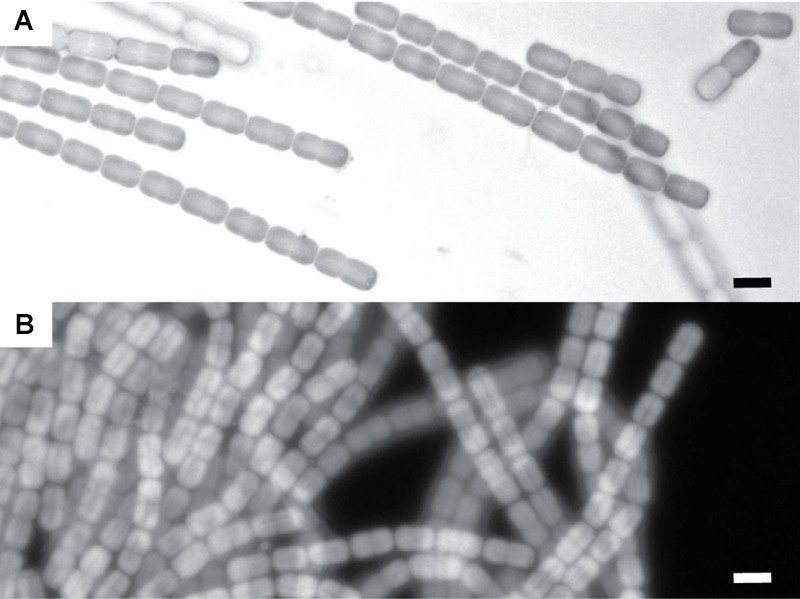
**Microscopic images of the cyanobacteria dominating the cyanobacterial mats in the Frasassi sulfidic springs. (A)** Light microscope image. **(B)** Auto-flourescence image (Excitation maximum: 547 nm, Emission > 590 nm). The scale bars are 5 μm.

The examined subsample of the cyanobacterial layer was dominated by a single filamentous cyanobacterium with a diameter of ∼4 μm (**Figure 3**). Besides this cyanobacterial morphotype we only observed unicellular non-pigmented prokaryotes (data not shown).

### Microsensor Measurements

#### Oxygenic and Anoxygenic Photosynthesis

**FIGURE 4:**
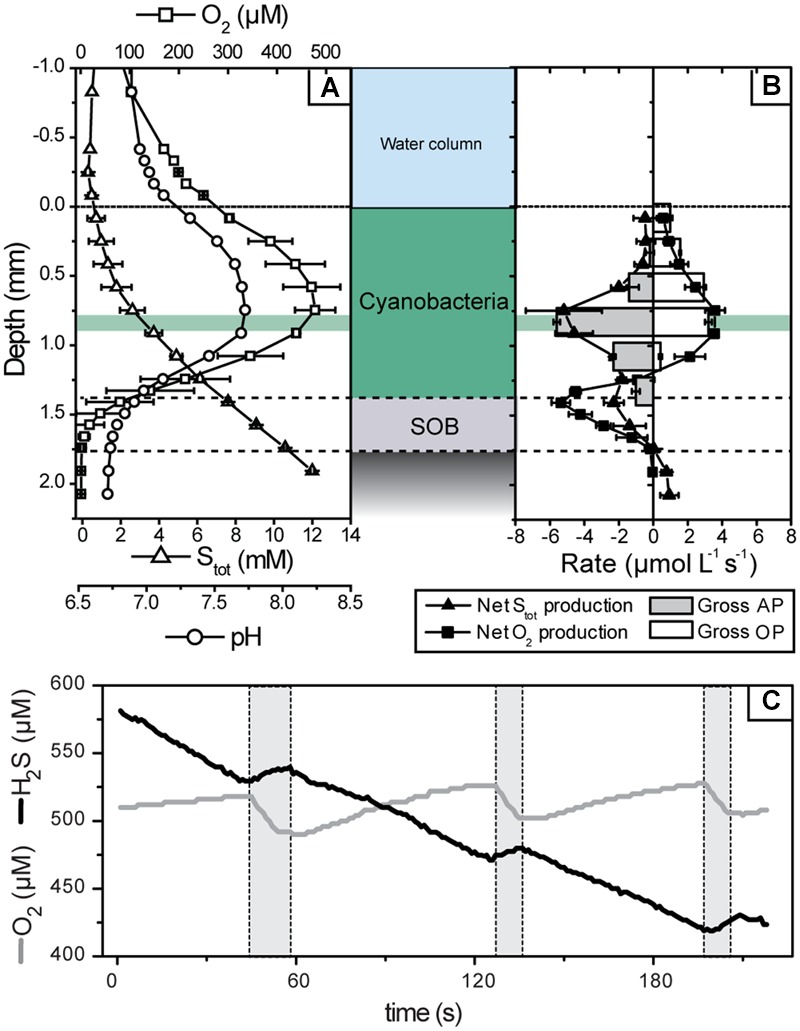
**Microsensor measurements of pH, H_2_S, and O_2_ concentrations in the mat at an incident irradiance of 339 μmol photons m^-2^ s^-1^. (A)** Steady-state concentration depth profiles. Error bars are standard deviation calculated from profiles measured before and after light–dark shift measurements (*n* = 4). **(B)** Net (symbols) and gross (bars) volumetric rates of OP and AP in the mat. Net rates were calculated from the depth profiles shown in **(A)** and then averaged (error bars are standard deviation), while gross rates were calculated based on the dynamics of O_2_ and H_2_S concentrations and pH measured in the mat during light–dark transitions. Note that the negative of the gross AP rate is depicted in **(B)** because it was measured as the rate of light-driven S_tot_ consumption. An example of these dynamics, measured at a depth with the highest activity (0.83 mm; marked by green horizontal area in **A,B**) during light (no shading) and dark (gray shading) intervals, is shown in **(C)**.

**FIGURE 5:**
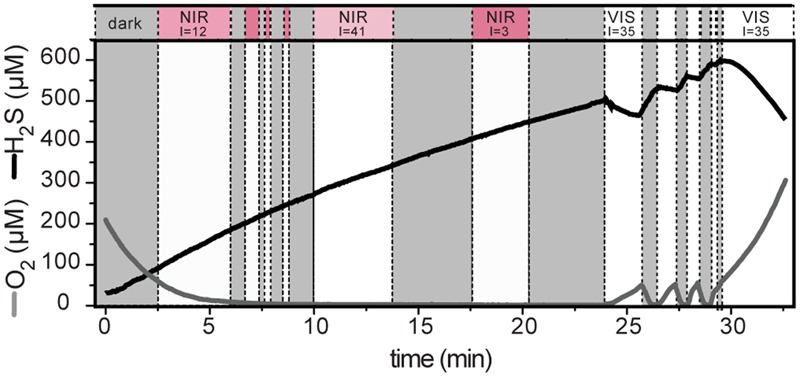
**Light-induced dynamics of H_2_S and O_2_ concentration measured at 0.83 mm depth in the cyanobacterial layer.** The locally available irradiance (I; in μmol photons m^-2^ s^-1^) is indicated above the white areas. The irradiance was either mainly in the visible part of the spectrum (VIS) or in the near infrared (NIR) range (1:3 mixture of photon fluxes with emission maxima at 740 and 810 nm). Shaded areas indicate darkness.

Upon illumination with visible light, total sulfide concentration in the cyanobacterial layer immediately decreased and O_2_ concentration increased, suggesting AP and OP, respectively. This was confirmed by detailed measurements of the local volumetric gross rates of AP and OP using the light–dark shift approach (**Figure 4**). An instantaneous increase of H_2_S and decrease of O_2_ upon darkening (e.g., **Figure 4C**), synonymous with the simultaneously active AP and OP, was detectable until about 1.4 mm depth (**Figure 4B**). In contrast to visible light, light in the near infrared region of the spectrum, which specifically targets bacteriochlorophylls of obligate anoxygenic phototrophs, did not induce sulfide consumption at any depth (**Figure 5**). Thus, all rates of sulfide-driven AP presented here were exclusively assigned to cyanobacteria.

**FIGURE 6:**
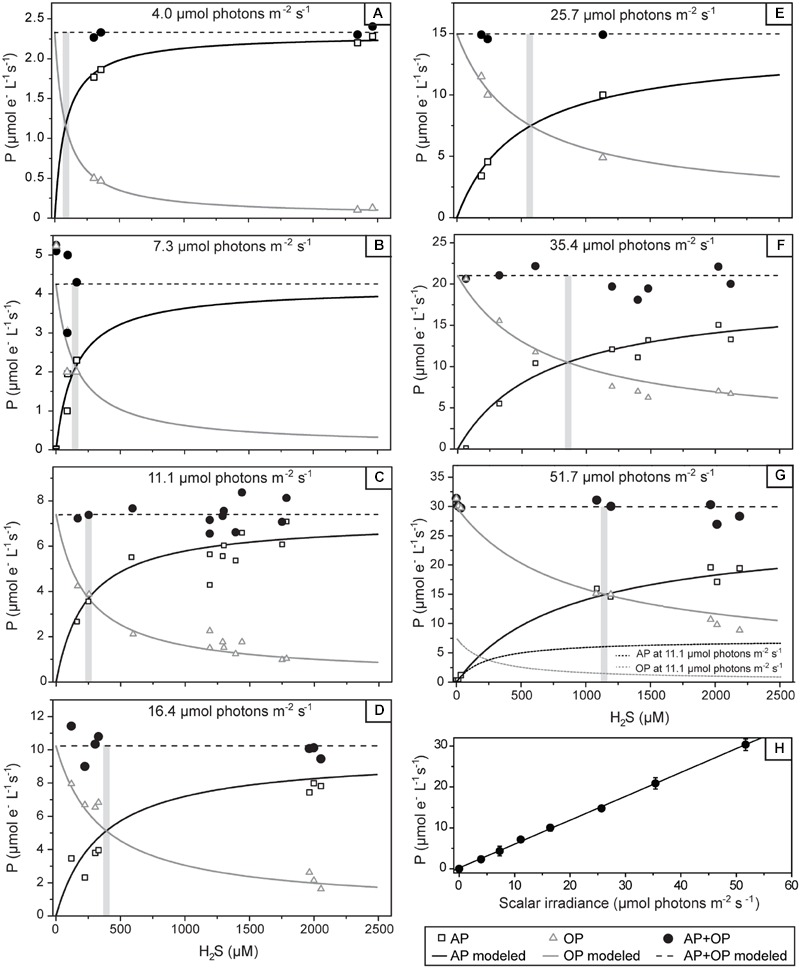
**Volumetric rates of gross photosynthesis in the studied cyanobacterium as a function of the H_2_S concentration and the locally available scalar irradiance.** The local scalar irradiance was derived from the depth profile shown in **Figure 2B**. Symbols show the measured rates of anoxygenic (AP) and oxygenic (OP) photosynthesis and their sum (AP+OP), all expressed in μM electrons s^-1^. Lines in **(A–G)** show the best fit of the results by the numerical model based on the scheme shown in **Figure 1**. The threshold H_2_S concentration above which OP exceeds AP is indicated by the shaded area. Modeled rates for 11.1 μmol photons m^-2^ s^-1^ are shown as dotted lines in **(G)** to allow for a direct comparison to the rates measured at 51.7 μmol photons m^-2^ s^-1^. In **(H)** the total photosynthetic electron transport rates in the studied cyanobacterium as a function of the locally available scalar irradiance are shown. Values are the mean (symbols) and standard deviations (error-bars) derived from all values given in **(A–G)**. Line shows the fit of the data-points by linear regression (*R* = 0.9994, *P* < 0.0001).

The parallel light–dark shift measurements of H_2_S and O_2_ concentration revealed that the gross rates of AP and OP were highest at 0.83 mm depth (**Figure 4B**). Scalar irradiance at this depth was about 10% of the surface value (**Figure 2B**). The detailed measurement of gross AP and OP at this depth of highest photosynthetic activity revealed that both types of photosynthesis (anoxygenic and oxygenic) were simultaneously performed over the complete range of light intensities and H_2_S concentrations tested (**Figure 6**). At a given local irradiance, the gross rates of AP increased with increasing H_2_S concentration, while the gross rates of OP decreased (**Figure 6**; open symbols). However, the total photosynthetic electron transport rate, i.e., the sum of electron transport rates due to OP and AP, did not depend on H_2_S (**Figure 6**; closed symbols). Light intensity affected the partitioning between the photosynthetic modes, which was most apparent in the light dependency of the initial slopes of OP decrease and AP increase with H_2_S concentration (**Figure 6**) and the light-dependency of the threshold H_2_S concentration above which AP became dominant over OP (**Figure 6**). The total electron transport rate increased linearly in the studied range of the locally available light (**Figure 6H**). In summary, both H_2_S concentration and light intensity regulated the partitioning between OP and AP, while the total electron transport rate was dependent only on light. Importantly, even at very low local light intensities (∼4 μmol photons m^-2^ s^-1^) and high H_2_S concentrations (>2.4 mM) we always observed simultaneous OP and AP and never only AP (**Figure 6**).

We applied a model of the photosynthetic electron transport chain (**Figure 1**) to fit our data, assuming that the single morphotype shown in **Figure 3** is one strain that is fully responsible for the observed rates. We found an excellent agreement between the measured and predicted rates of OP and AP and their light- and H_2_S-dependent partitioning (compare symbols with lines in **Figures 6A–G**). To fit the data we must assume that the apparent affinity of SQR to H_2_S is very low (K_M_ > 1 mM) and that the apparent affinities of SQR and PSII to oxidized PQ are very similar (K_PSII_/K_SQR_ ≈ 1).

#### Non-photosynthetic Sulfide Consumption

**FIGURE 7:**
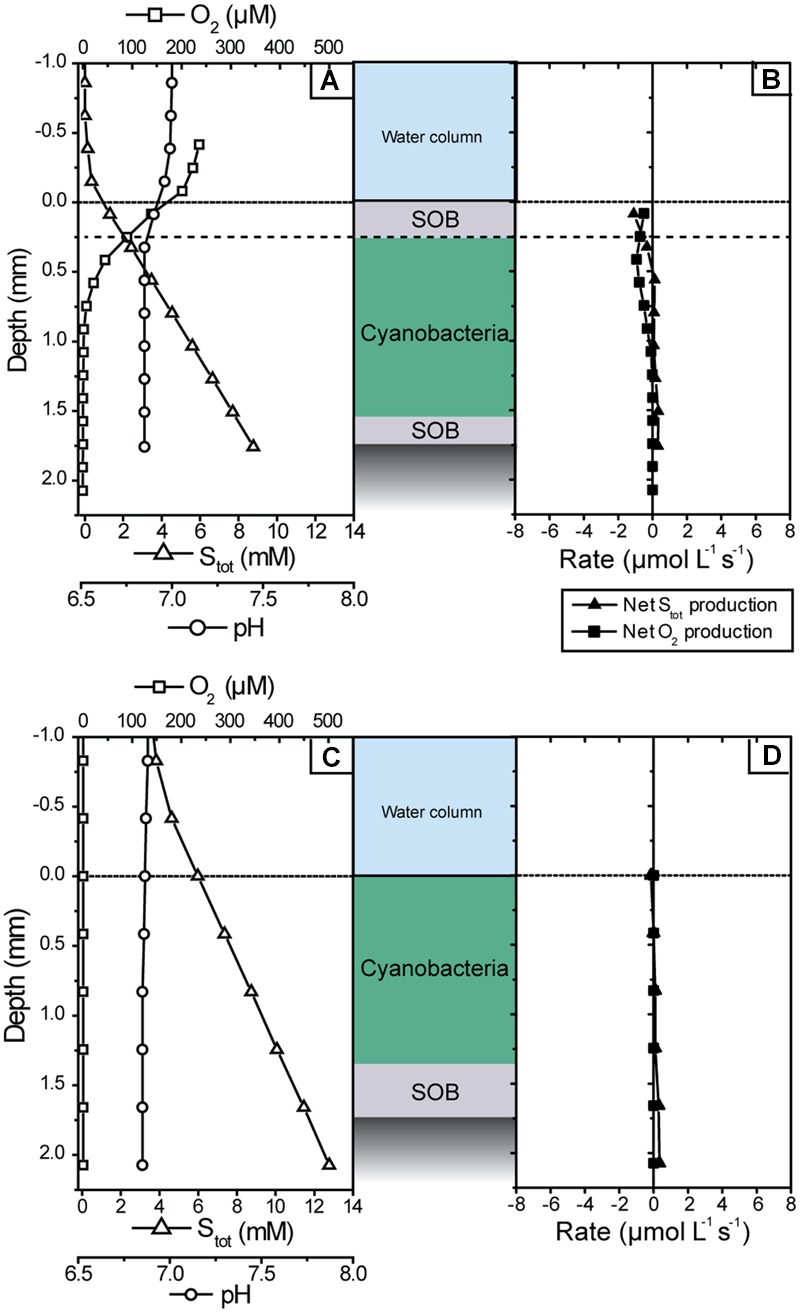
**Microsensor measurements of pH, O_2_ and total sulfide concentration measured in the cyanobacterial mat in the dark** shortly after sampling **(A,B)** and after all measurements in the light were completed **(C,D)**. **(A,C)** Show steady-state depth profiles of concentrations while **(B,D)** show the corresponding volumetric net conversion rates of S_tot_ and O_2_.

Microsensor depth profiles measured in the dark revealed that there were no significant non-photosynthetic sulfide sinks in the cyanobacterial layer of the mat. In the presence of oxygen in the water column, sulfide and oxygen showed opposing gradients in the uppermost layer (**Figure 7A**), suggesting chemosynthetic sulfide oxidation. However, the volumetric rates of sulfide consumption were very low and restricted to the top 0.1–0.2 mm, consistent with the slightly whitish appearance of the mat due to the low abundance of chemosynthetic large filamentous SOB. In contrast, O_2_ consumption, although low, extended down to ∼0.8 mm (**Figure 7B**), indicating that the main O_2_ sink in the mat in darkness was aerobic respiration, e.g., by the cyanobacteria or heterotrophic bacteria. Upon turn of the system to anoxia, there was no detectable sulfide consumption at any depth (**Figures 7C,D**).

Upon illumination, the net volumetric rates of S_tot_ consumption in the cyanobacterial layer closely matched the gross rates of AP (**Figure 4B**), confirming that non-photosynthetic sulfide consumption was insignificant. Likewise, oxygen consumption rates in the cyanobacterial layer were below detection limit (**Figure 4B**). Instead, the oxygen produced by OP was consumed in the thin SOB layer underneath the photosynthetically active zone (below 1.4 mm depth; **Figure 4B**). In this layer also net sulfide consumption occurred (**Figure 4B**) while we did not observe instantaneous changes in sulfide concentration in response to abrupt changes in illumination (see Oxygenic and Anoxygenic Photosynthesis). This suggests that AP was insignificant while aerobic sulfide oxidation was the dominant sink of both sulfide and oxygen in the SOB layer during illumination.

## Discussion

The cyanobacterial layer in the studied mat performed OP and AP simultaneously over a wide range of H_2_S concentrations and light intensities. The regulation of the total photosynthetic electron transport given by the sum of electron transports driven by AP and OP was astonishingly simple. Specifically, the total electron transport rate increased linearly with irradiance and did not depend on H_2_S at all (**Figure 6H**). The partitioning between oxygenic and anoxygenic modes seemed more complex as it was controlled by both H_2_S levels and light intensities (**Figures 6A–G**).

Using our numerical model of the electron transport reactions, we found that the observed activity patterns in this mat can be explained by kinetic controls. Thus, synthesis or degradation of cell components, such as pigments and photosystems, is not needed as an explanation for the complex light- and H_2_S-dependency of photosynthetic rates. We furthermore exclude changes in the abundance of cell components as an explanation for our data, because all our measurements were performed within a short time (∼6 h) and because the observed pattern emerged even though we did not follow a strict order (e.g., either gradually increasing or gradually decreasing) of light intensities and H_2_S concentrations. Also, we repeated the first measurements at the end of our experiments and showed that the physiologic properties of the mat, i.e., the overall photosynthetic rate and the ratio between AP and OP, had not significantly changed. We therefore conclude that the adjustment of rate and rate partitioning in response to the momentary microenvironmental light and H_2_S conditions were instantaneous and the result of kinetic control.

**FIGURE 8:**
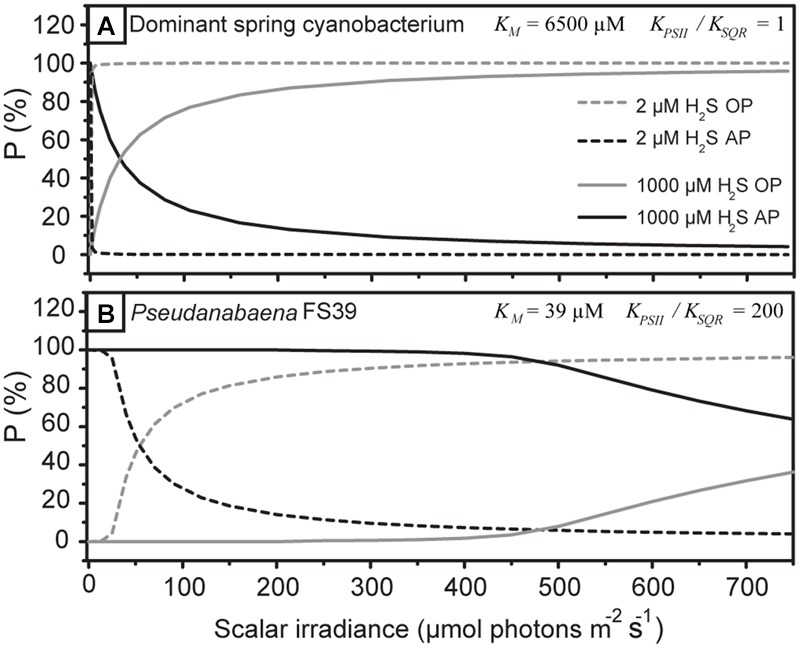
**Partitioning between AP and OP predicted by the model shown in **Figure 1** for the cyanobacterium studied here (A)** and for *Pseudanabaena* str. FS39 **(B)** at low and high H_2_S concentrations.

The specific H_2_S and light dependent partitioning between OP and AP can be explained by considering that the light-driven electron transport from PSII and the H_2_S-driven electron transport from SQR intersect in the plastoquinone (PQ) pool (**Figure 1**). The electron transport rates are controlled by the affinities governing the rates of PQ reduction by PSII (k_oxy_ in **Figure 1**) and SQR (k_anoxy_). We determined that the apparent affinity of SQR to H_2_S has to be very low (K_M_ > 1) (see notation in **Figure 1**). More importantly, the apparent affinities of SQR and PSII to PQ are in the same range. This means that PSII and SQR can compete equally well for oxidized PQ. As a consequence, OP is performed simultaneously with AP irrespective of the light intensity and H_2_S concentration (**Figure 8A**), even at H_2_S concentrations that are higher than ever observed *in situ* in the naturally occurring mats (Klatt et al., 2016).

We have observed such kinetic regulation mechanism previously in another cyanobacterium: *Pseudanabaena* str. FS39, a versatile cyanobacterium enriched from the same environment (Klatt et al., 2015a). As the apparent affinities of SQR and PSII toward PQ, however, fundamentally differ from the cyanobacterium studied here, a completely different H_2_S- and light-dependent activity pattern emerges (**Figure 8B**). In *Pseudanabaena* str. FS39 the apparent affinity of SQR to H_2_S is very high, with the corresponding K_M_ at least two orders of magnitude lower than in the cyanobacterium studied here. Moreover, in *Pseudanabaena* str. FS39 the affinity of SQR to oxidized PQ is at least two orders of magnitude higher than that of PSII (K_PSII_/K_SQR_ ≥ 100). In other words, OP is kinetically outcompeted by AP. This is manifested by the fact that OP is only performed in addition to AP when H_2_S is limiting SQR activity. As a consequence, *Pseudanabaena* str. FS39 switches from exclusive AP to simultaneous AP and OP at substantially lower H_2_S concentrations and/or higher light intensities than the cyanobacterium studied here (compare **Figures 8A,B**).

The striking differences between the affinities of SQR to PQ and H_2_S in *Pseudanabaena* str. FS39 and the dominant spring cyanobacterium suggest that their SQRs belong to different enzyme structure classes (Gregersen et al., 2011). The affinities of PSII to PQ also differ among the two cyanobacteria. Protein D1, which is a core component of the photosystem II reaction center, shapes the kinetics of quinone reduction. Different isoforms of this protein exist and are expressed dependent on oxygen concentration and light (Cardona et al., 2015). We therefore suggest that *Pseudanabaena* str. FS39 and the cyanobacterium studied here might employ fundamentally different types of D1. Thus, there might be a relationship between the type of D1 and regulatory mechanisms for AP, which might have far-reaching implications for research on the evolution of AP in cyanobacteria. The phylogeny of genes encoding for the well-conserved D1 proteins was successfully used to reconstruct their evolutionary history (Cardona et al., 2015). Such reconstruction is challenging when based only on SQR genes due to their history of intensive lateral gene transfer (Theissen et al., 2003). Genes encoding for several isoforms of D1 have recently been detected in the genome of *Geitlerinema* sp PCC 9228 (formerly *Oscillatoria limnetica*) (Grim and Dick, 2016), the most intensively studied photosynthetically versatile cyanobacterium, which further underlines the intriguing question if usage of different D1 types might be an important tool for cyanobacteria in the regulation of AP.

It is very unlikely that the observed activity pattern emerged from the simultaneous activity of a mix of several physiologically distinct cyanobacterial species or strains. The studied mats were dominated by a single cyanobacterial morphotype. The complex experimental data could be fitted by a simple kinetic model, thus by one kinetic expression per reaction step. Any residual uncertainty does, however, not invalidate the general conclusion that the most successful cyanobacterial population in the sulfidic spring can perform simultaneous OP and AP over a complete diurnal cycle unless H_2_S becomes entirely depleted within the photosynthetically active zone.

The range of H_2_S concentrations and irradiances over which OP and AP are performed concurrently in the studied mats is substantially wider than in *Pseudanabaena* FS39. In fact, it is even exceptional compared to other previously studied versatile cyanobacterial strains. It was shown that *Geitlerinema* PCC9228, for instance, performs simultaneous OP and AP only below a 80 μM H_2_S concentration threshold when exposed to 400 μmol photons m^-2^ s^-1^ (Cohen et al., 1986). Cohen et al. (1986) also report that in an *Oscillatoria* sp. from Stinky Hot Springs and in *Microcoleus chtonoplastes* (*Geitlerinema*) from Solar Lake, AP and OP occur concurrently below 550 μM H_2_S and 1000 μM H_2_S, respectively, at the same light intensity. For the case of the Frasassi sulfidic spring cyanobacterium, our model predicts that the OP:AP ratio would still be 0.9 at 1000 μM H_2_S and that exclusive AP is not possible at all at around 400 μmol photons m^-2^ s^-1^.

This astonishing coverage of H_2_S concentrations and light intensities inspires an intriguing question: what is the advantage of simultaneous AP and OP in general, and specifically in the Frasassi sulfidic springs? As opposed to obligate anoxygenic phototrophs, both obligate oxygenic phototrophs and versatile cyanobacteria are generally never limited by electron donor availability. There is, however, a major difference between versatile and obligate oxygenic cyanobacteria: AP is driven by only one photosystem (PSI), whereas OP is driven by two photosystems (PSI+PSII). Thus, theoretically, the photon flux required to drive a certain electron transport and growth rate is twice as large for OP as for AP. Due to this lower energy demand of AP, one could expect that AP is favorable for cyanobacteria when H_2_S is not limiting. However, this expected advantage of AP seems not to necessarily hold true in versatile cyanobacteria. For the studied cyanobacterium and also for *Pseudanabaena* str. FS39 we have shown that the total electron transport rate driven by a specific photon flux is independent of H_2_S concentrations and the photosynthetic mode. A constant electron transport implies that the light energy harvested in PSII is basically wasted during AP. This is because only PSI is involved in anoxygenic electron transport and photons harvested in PSII are therefore not efficiently used to drive photochemical reactions. AP would only be advantageous if the excitation energy were transferred from PSII to PSI or if the photosystem stoichiometry changed (Klatt et al., 2015a). This does not seem to occur in the cyanobacterium dominating the Frasassi sulfidic springs as the sum of photosynthetic electron transport rates is constant. Thus, AP does not appear to provide any energetic advantage to this phototroph.

Considering that quantum efficiency in cyanobacteria can be constant irrespective of the photosynthetic mode, cyanobacteria that perform only one mode of photosynthesis therefore can, from a thermodynamic perspective, theoretically be as successful as versatile cyanobacteria. However, obligate oxygenic phototrophs in the Frasassi sulfidic springs (e.g., *Planktothrix* str. FS34, Klatt et al., 2015b) do not seem to dominate the photosynthetic community. Similarly, cyanobacteria that would perform only AP for most of the diurnal cycle (e.g., *Pseudanabaena* str. FS39, Klatt et al., 2015a, **Figure 8B**) are not abundant. Instead, the key to success appears to lie in the simultaneity of both photosynthetic modes.

Production of oxygen in the presence of sulfide is tied to severe toxification risks by reactive oxygen species (ROS) generation (Latifi et al., 2009; Hoffman et al., 2012). A counterbalancing advantage of such adaptation strategy remains mysterious to us – especially in the modern world where potential competitors for space and resources [e.g., SOB and obligate anoxygenic phototrophs (Klatt et al., 2016)] as well as microorganisms that might affect this competition via feedback mechanisms on the S- and C-cycle (e.g., sulfate reducers) have developed sophisticated strategies to cope with fluctuating oxygen and sulfide concentrations (e.g., Blankenship and Matsuura, 2003; Baumgartner et al., 2006; Berghoff et al., 2011). We can therefore only speculate that the advantage of versatility might be understood by considering that cyanobacteria are not an independently operating entity but almost always rely on the interaction with other microbes, which is most apparent in the frequent failure of axenic cultivation of cyanobacteria. In this scenario a close beneficial interaction with aerobs and/or oxidizers of the products of cyanobacterial AP, i.e., intermediately reduced sulfur compounds (Cohen et al., 1975; de Wit and van Gemerden, 1987; Rabenstein et al., 1995), is plausible and represents a hypothesis that warrants testing.

While the advantage of continuous O_2_ production and sulfide consumption at virtually any light intensity and H_2_S concentration in contemporary ecosystems remains to be understood, the capability to perform OP whenever there is a glimpse of light might have provided the ancestors of extant cyanobacteria with a crucial advantage in the microbial mats that covered Earth’s costal oceans for much of the Proterozoic and likely already during the Archean. These mats represent the stage for the evolution and proliferation of OP (Buick, 2008; Lyons et al., 2014), a process which must have been a catastrophe for microbial life in an anaerobic world but also triggered the co-evolution of aerobic life. Thus, even the earliest aerobic life-style must have been able to thrive in the fluctuating redox conditions of microbial mats. Overall, our study emphasizes that the evolution of cyanobacterial adaptations strategies and the resulting cyanobacterial activity, i.e., both the gross and net O_2_ production rates in microbial mats, were tightly coupled to the evolution of Earth’s redox environment (that is, the availability of alternative electron donors for photosynthesis), the energy supply in the form of light and chemical energy and likely the co-evolution of competitive and beneficial microbial interactions.

## Author Contributions

JK, DB, SH, and LP made substantial contributions to the conception and the design of the study, the acquisition of data, and the analysis and interpretation of data; JK, DB, SH, and LP participated in drafting the article and revising it critically for important intellectual content; JK, DB, SH, and LP gave final approval of the version to be submitted. JK, DB, SH, and LP agreed to be accountable for all aspects of the work in ensuring that questions related to the accuracy or integrity of any part of the work are appropriately investigated and resolved.

## Conflict of Interest Statement

The authors declare that the research was conducted in the absence of any commercial or financial relationships that could be construed as a potential conflict of interest.
